# Comparison of Day-Specific Serum LH, Estradiol, and Progesterone with Mira^TM^ Monitor Urinary LH, Estrone-3-glucuronide, and Pregnanediol-3-glucuronide Levels in Ovulatory Cycles

**DOI:** 10.3390/medicina60081207

**Published:** 2024-07-26

**Authors:** Stephen J. Usala, David D. Vineyard, Maria Kastis, A. Alexandre Trindade, Harvinder Singh Gill

**Affiliations:** 1 Department of Internal Medicine, Texas Tech University Health Sciences Center, Amarillo, TX 79106, USA; 2 Department of Obstetrics and Gynecology, Texas Tech University Health Sciences Center, Amarillo, TX 79106, USA; david.vineyard@ttuhsc.edu; 3 School of Medicine, Texas Tech University Health Sciences Center, Lubbock, TX 79430, USA; maria.kastis@ttuhsc.edu; 4 Department of Mathematics and Statistics, Texas Tech University, 1108 Memorial Circle, Lubbock, TX 79409, USA; alex.trindade@ttu.edu; 5 Harvinder Singh Gill, Department of Chemical and Biomolecular Engineering, North Carolina State University, Raleigh, NC 27695, USA; hsgill2@ncsu.edu

**Keywords:** fertility tracking, fertile window, NFP, FABM, Mira, fertility indicator equation, AUC algorithm, E3G, PDG

## Abstract

*Background and Objectives*: Fertility tracking apps and devices are now currently available, but urinary hormone levels lack accuracy and sensitivity in timing the start of the 6-day fertile window and the precise 24 h interval of transition from ovulation to the luteal phase. We hypothesized the serum hormones estradiol (E2) and progesterone (P) might be better biomarkers for these major ovulatory cycle events, using appropriate mathematical tools. *Materials and Methods*: Four women provided daily blood samples for serum E2, P, and LH (luteinizing hormone) levels throughout their entire ovulatory cycles, which were indexed to the first day of dominant follicle (DF) collapse (defined as Day 0) determined by transvaginal sonography; therefore, ovulation occurred in the 24 h interval of Day −1 (last day of maximum diameter DF) to Day 0. For comparison, a Mira^TM^ fertility monitor was used to measure daily morning urinary LH (ULH), estrone-3-glucuronide (E3G), and pregnanediol-3-glucuronide (PDG) levels in three of these cycles. *Results*: There were more fluctuations in the Mira^TM^ hormone levels compared to the serum levels. Previously described methods, the Fertility Indicator Equation (FIE) and Area Under the Curve (AUC) algorithm, were tested for identifying the start of the fertile window and the ovulation/luteal transition point using the day-specific hormone levels. The FIE with E2 levels predicted the start of the 6-day fertile window on Day −7 (two cycles) and Day −5 (two cycles), whereas no identifying signal was found with E3G. However, both pairs of (E2, P) and (E3G, PDG) levels with the AUC algorithm signaled the Day −1 to Day 0 ovulation/luteal transition interval in all cycles. *Conclusions*: serum E2 and (E2, P) were better biomarkers for signaling the start of the 6-day fertile window, but both Mira^TM^ and serum hormone levels were successful in timing the [Day −1, Day 0] ovulatory/luteal transition interval. These results can presently be applied to urinary hormone monitors for fertility tracking and have implications for the direction of future fertility tracking technology.

## 1. Introduction

The use of fertility tracking apps and associated fertility tracking monitors linked to apps is now widespread [1,2,3,4,5,6,7,8]. One detailed review concluded women use this technology for various reasons which can change with time—self-knowledge, to conceive, as a method of birth control, and to assess fertility treatments [9].

To meet women’s great demand for accurate and reliable fertility tracking technology, fertility devices based upon measurement of urinary hormones, luteinizing hormone (LH), estrone-3-glucuronide (E3G), and pregnanediol-3-glucuronide (PDG) have been extensively researched, developed, and marketed [1,2,3,4,5,6,7,8,9,10,11,12,13,14,15]. The timing of the fertile window is the critical information required to avoid pregnancy and to conceive [16]. The day-specific probabilities of pregnancy throughout the menstrual cycle have been well established in multiple studies [17,18,19]. These studies have proven that the probability of conception is very low outside a 6-day window, Day −5 to Day 0, where Day 0 is the day of ovulation and Day −1 to Day −5 are the five preceding days. The days of highest fertility are generally one to two days before ovulation, Day −2 and −1, whereas the day of ovulation is actually a day of relatively low probability of conception [17,18,19].

However, the fertility tracking devices based upon these urinary hormones have distinct limitations especially for birth control purposes. Ovulation occurs approximately 24 h after the urine LH (luteinizing hormone) peak [13,14]. Urinary E3G levels during the fertile window cover a wide range: there is a considerable standard deviation from the day-specific means and, therefore, absolute levels cannot be used to identify the start of the 6-day fertile window [15,20,21,22]. Both the Mira^TM^ and Inito^TM^ monitor systems, which quantify E3G levels, give fluctuating E3G levels preceding the start of and during the fertile window [4,15]. Only 75% of women had adequate warning of the 6-day fertile interval to avoid pregnancy using the E3G-based ClearBlue Fertility Monitor^TM^ [15]. Another fertility tracking monitor, the Proov Complete^TM^, provided an average of 2.68 days between the E3G rise and the LH surge, which is unsuitable for birth control applications [3]. Furthermore, these tracking systems lack precision in identifying the day of transition to the luteal phase, which shortens the ‘safe’ infertile luteal interval for sexual intercourse. Multiple studies set a PDG threshold of 5 mcg/mL for entry into this safe infertile luteal phase, which appears to allow a corresponding length of 8.8 days on average [11,12,23,24,25,26]. In summary, the present fertility tracking technology using the urinary hormones E3G and PDG does not reliably signal the start of the 6-day fertile window, does not optimally signal the start of the luteal safe days, and, therefore, needs improvement for natural family planning.

We hypothesized that serum levels of estradiol (E2) and progesterone (P)—or rather the rate of change of (E2, P)—might enable the quantification of time-of-cycle, that is, signaling for the start of the 6-day fertile window and the day of ovulation/transition to the luteal phase. To answer this question, individual cycles with day-specific E2 and P levels, not mean day-specific levels, are necessary. Day-specific E2 and P levels for individual cycles are rare to non-existent in the literature or on internet searches, although recently a publication with normalized day-specific plots for 20 subjects was presented to understand menstrual cycle hormonal variabilities [27]. As these authors state: ‘obtaining daily blood samples… over the entire menstrual cycle is expensive and difficult to implement in practice’ [27].

Here, we present the daily serum E2, LH, and P levels for four ovulatory cycles and, in addition, the daily urinary E3G, LH, and PDG levels from three of these cycles obtained using the popular Mira^TM^ fertility tracking monitor. Day-specific E2 and P, and E3G and PDG, levels were rendered by indexing to the day of transition from the maximum dominant follicle (DF), denoted Day −1, to the first day of DF collapse, denoted Day 0; ovulation must have occurred in the 24 h interval between Day −1 and Day 0. These day-specific serum E2, P and urinary E3G, PDG levels were then used to test previously published algorithms developed for signaling the 6-day fertile window and the day of transition to the luteal phase [22,28,29].

## 2. Materials and Methods

### 2.1. Subjects

Four women were recruited for one cycle each (cycles 1Y1, 2Y1, 4Y1, and 6Y1) for this study. They were 27–32 years of age, had a BMI of 18.6–26.2, reported regular cycles of 25–28 days for the previous six months, and were not on any hormonal contraceptives. This study was approved by the TTUHSC Amarillo IRB #A23-4337. After informed consent was obtained the patients supplied blood by venipuncture every morning at the TTUHSC Amarillo clinical center, non-fasting, 8:30 a.m.–11:30 a.m., starting calendar day 1 (CD 1) of their cycle until the beginning of menses of the next cycle. In addition, prior to the blood draws, subjects 2Y1, 4Y1, and 6Y1 were provided Mira fertility monitors with urinary LH, E3G, and PDG wands to gain familiarity with the device. They started to record data for the study using the first morning urine the day after menses ended for the duration of the blood draws.

### 2.2. Transvaginal Sonography

Transvaginal sonography every 10:00 a.m.–noon was started seven days before the estimated day of ovulation and continued until two days of dominant follicle (DF) collapse. Transvaginal images and data storage were performed with a Philips EPIQ 7 ultrasound machine in the Department of Obstetrics & Gynecology, Texas Tech University Health Sciences Center (TTUHSC) Amarillo. Day 0, herein, is defined as the first day of DF collapse, and Day −1 is, herein, defined as the last day of the DF where the maximum diameter was observed. Therefore, the release of an ovum (ovulation) must have occurred in the 24 h interval defined by Day −1–Day 0. For this study, 34 transvaginal ultrasound examinations were completed for the four subjects, comprising 475 saved images. These examinations were conducted in accordance with the practice parameters for the performance of a focused ultrasound examination in reproductive endocrinology and female infertility, as published by AIUM (American Institute of Ultrasound in Medicine) [30].

All follicles were measured in two perpendicular dimensions and the means were recorded. The dominant follicle, the one most responsive to the hormonal mediators of follicle development and destined for ovulation rather than atresia, is also the largest of the follicles that develops during this phase. Ovulation was signaled by a decrease in size of the dominant follicle and changes in the cyst wall.

### 2.3. Daily Serum Samples

Only one blood draw was missed (4Y1, Day −11) and only one urine measurement was missed (6Y1, Day +3), which did not affect the interpretation of the results. Serum samples from the blood draws were aliquoted and stored at −80 °C. Serum E2, P, and LH were obtained in the Panhandle Reproductive Research Lab, the clinical laboratory for the TTUHSC Amarillo clinics. They were certified by the College of American Pathologists (CAP) and CLIA. Subject serum specimens were assayed with the Abbott Architect ci4100.

The serum LH, E2, and P and the urinary LH (ULH), E3G, and PDG were all indexed to Day 0 to render day-specific levels for cycles 1Y1, 2Y1, 4Y1, and 6Y1.

### 2.4. Fertility Indicator Equation (FIE) and Area Under the Curve (AUC) Algorithms

Previously devised algorithms, the Fertility Indicator Equation, FIE, and the Area Under the Curve, AUC, were tested with the day-specific E2, P and E3G, PDG levels [22,28,29]. FIE was applied to the day-specific E2 and day-specific E3G levels to inform on the start of the fertile window, in particular, and also the day of ovulation/transition to the luteal phase. FIE (E2) and FIE (E3G) were formulated as follows. For every day, D, of the cycle, the Delta function values were first calculated:

Delta (D-1) = (E2 or E3G level on Day, D-1 − E2 or E3G level on Day, D-2)/(E2 or E3G level on D-2)

Delta (D) = (E2 or E3G level on Day, D − E2 or E3G level on Day, D-1)/(E2 or E3G level on D-1)

FIE on D was then calculated as the Delta product × 100:

Delta (D) *×* Delta (D-1) × 100

The sign of FIE is determined as follows:

+Delta (D-1) *×* +Delta (D) = +FIE

−Delta (D-1) *×* −Delta(D) = −FIE

However, they are indeterminate (‘ind’) if the product is (+) (−), (−) (+)—there is uncertainty in the direction of change.

The AUC algorithm, which was previously developed using urinary E3G and PDG levels measured by radioimmunoassay from M.E. Alliende et al. [21,22], was tested using the day-specific E2, P and E3G, PDG levels. GraphPad Prism version 10.2.3 for Windows v11, GraphPad software, San Diego, CA, USA (www.graphpad.com, accessed on 25 May 2024), was used for AUC computations. The AUC algorithm is based upon computing the daily Area Under the Curve for E2 and P as well as AUCE2 and AUCP, respectively, in a progressive manner from the start of the cycle, which leads to the calculation of a day-specific ratio, AUCE2/AUCP. In a similar fashion, the day-specific ratio, AUCE3G/AUCPDG, can be determined. Finally, the metric examined was the difference in this ratio (i.e., for AUCE2/AUCP or AUCE3G/AUCPDG) between two successive days, which renders the day-specific difference, Delta(AUCE2/AUCP) or Delta(AUCE3G/AUCPDG).

## 3. Results

### 3.1. Day-Specific Serum LH, E2, and P Levels Compared with Day-Specific Urinary LH, E3G, and PDG Levels

Four subjects provided daily blood during cycles 1Y1, 2Y1, 4Y1, and 6Y1 for serum LH, E2, and P levels. The subjects had a history of regular periods, and these study cycles were of normal length (Table 1). Daily morning urine for three of these cycles, 2Y1, 4Y1, and 6Y1, except during days of menses (2Y1, four days; 4Y1, five days; and 6Y1, seven days), were assayed with the Mira^TM^ fertility monitor for urine LH (ULH), E3G, and PDG levels. Daily morning periovulatory sonography was performed for seven days, 1Y1; nine days, 2Y1; nine days, 4Y1; and nine days, 6Y1. The release of an ovum, which is the time of ovulation, would, therefore, have occurred between Day −1, the last day of the DF at maximum diameter, and Day 0, the first day of DF collapse (Table 1). The day-specific serum and urinary hormone levels were indexed to Day 0, the first day of dominant follicle (DF) collapse.

**Figure 1:**
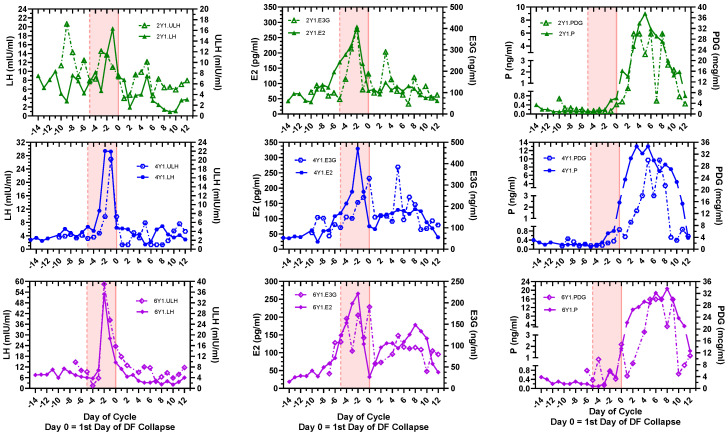
Comparison of day-specific serum LH, E2, and P levels versus day-specific urinary Mira^TM^ LH, E3G, and PDG levels for cycles 2Y1, 4Y1, and 6Y1. Subjects 2Y1, 4Y1, and 6Y1 provided daily morning blood for serum samples starting on the first day of menses until the beginning of the next menses. These subjects started daily urinary hormone measurements with the Mira^TM^ the day after menses ended until the start of the next cycle. Transvaginal sonography was performed daily for 7–9 days, encompassing the last day of the dominant follicle (DF) and the first day of DF collapse. The day-specific levels are indexed to the first day of DF collapse. The shaded interval (−5, 0) is the predicted 6-day fertile window. Important characteristics of the cycle are summarized in Table 1. E2: 1 pg/mL = 3.67 pmol/L; P: 1 ng/mL = 3.18 nmol/L; E3G: 1 ng/mL = 2.23 nmol/L; and PDG: 1 mcg/mL = 2.01 μmol/L.

The general configurations of the profiles for serum LH, E2, and P vs. ULH, E3G, and PDG were in overall agreement (Figure 1). The major events worthy of tracking for fertility are the fertile window, ovulation, and the transition to the luteal phase. The fertile window has been established to be a 6-day interval inclusive of the day of ovulation and five days prior [17,18,19]. Here, this interval is shown as Day −5 to Day 0 (pink region, Figure 1).

**Figure 2:**
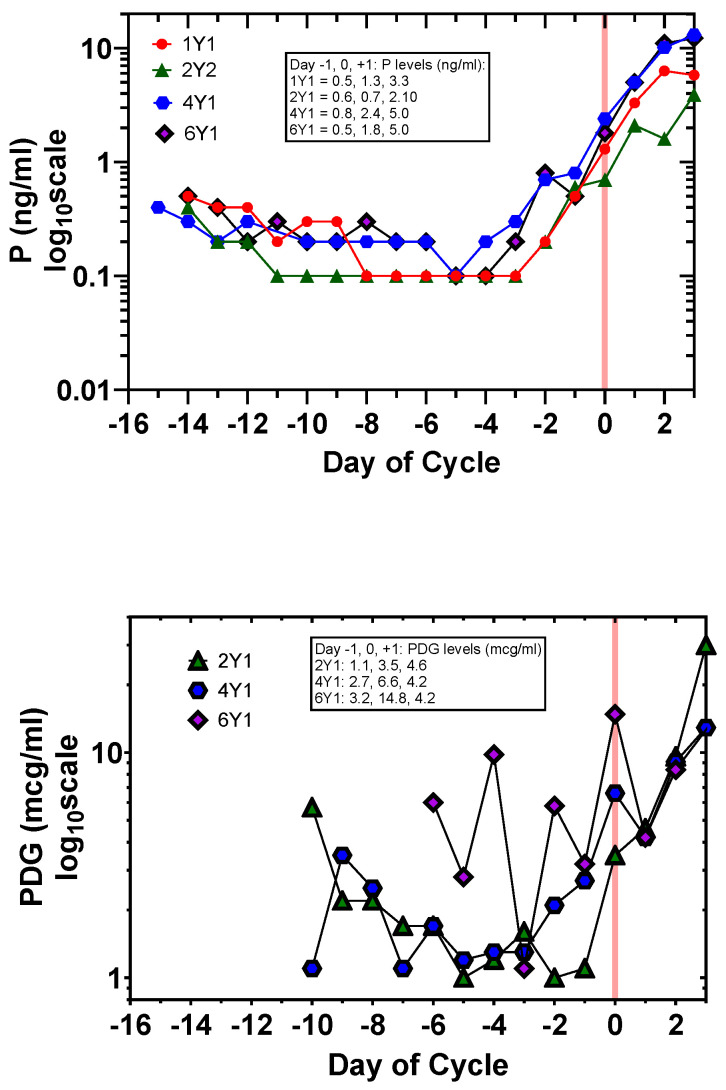
Comparison of high-resolution plots of day-specific P versus day-specific PDG for cycles 1Y1, 2Y1, 4Y1, and 6Y1. Serum P (cycles 1Y1, 2Y1, 4Y1, and 6Y1) and urinary PDG (2Y1, 4Y1, and 6Y1) are plotted on a log scale for the preovulatory interval to Day +3. Day 0 is the day of dominant follicle collapse (red line). There is an earlier and greater increase in serum P compared to PDG on the approach to Day 0 (Days −2, −1, and 0). P: 1 ng/mL = 3.18 nmol/L; and PDG 1 mcg/mL = 2.01 μmol/L.

Although the configurations of the plots were similar, serum E2 and P levels showed more accuracy and less fluctuation in terms of the timing and magnitude of the increases prior to the start of the fertile window and the time of ovulation, respectively (Table 1 and Figure 1 and Figure 2). There was an apparent rise in E2 levels at the start of the fertile window and an apparent rise in P levels near ovulation, both of which were measurable by mathematical analysis (following sections). In all four cycles, the serum E2 peak occurred on Day −1. Interestingly serum P showed a significant rise from the post-menses follicular phase on Day −2 for all three cycles. This contrasted with PDG where in two of three cycles a significant rise did not occur until Day 0.

**Table 1:** Key attributes of ovulatory cycles 1Y1, 2Y1, 4Y1, and 6Y1 ^a^.

	1Y1	2Y1	4Y1	6Y1
Dominant Follicle (DF) ^b^ Max Size (mm) (Day −1)	22.3	30.3	22.4	26.9
Cycle length (days)	25	27	28	27
LH peak day	−1	−1	−2, −1	−2
ULH peak day	-	−9, −3	−1	−1
Preovulatory E2 peak day	−2	−2	−2	−2
Preovulatory E3G peak day	-	−2	0	0
Preovulatory E2 range (pg/mL) (1 pg/mL = 3.67 pmol/L)	28.1–362.8	42.7–285.5	24.3–329.2	31–265.6
Preovulatory E3G range (ng/mL) (1 ng/mL = 2.23 nmol/L)	-	84.6–396.1	62.4–331.5	34.4–190.4

^a^ E2 and LH are serum levels; ULH (urinary LH) and E3G levels of morning urine were determined with the Mira^TM^ device for 2Y1, 4Y1, and 6Y1. ^b^ Transvaginal sonography was performed daily for a 7–9 day interval encompassing the last day of the dominant follicle (DF) (defined as Day −1) and the first day of DF collapse (defined as Day 0). In 1Y1, however, the sonography was stopped at a DF size of 22.3 mm (day of serum LH peak) and the day of collapse was inferred from this DF size and the following two serum progesterone levels. In addition, the serum progesterone levels for 2Y1, 4Y1, and 6Y1 all correlated with the day of DF collapse visualized by sonography (Figure 2).

### 3.2. Application of the Fertility Indicator Equation (FIE) to Signal the Fertile Window, Ovulation, and Luteal Phase Using Day-Specific E2 and Day-Specific E3G Levels

These day-specific E2 and E3G levels enabled testing of the mathematical formulation, the Fertility Indicator Equation (FIE), for the prediction of the days of the fertile window and day of ovulation and luteal transition [22,28,29]. The day-specific FIE was based upon the product of the relative change in the hormone level of two consecutive days with a sign (+, −, or 0) for confidence of direction. The day-specific FIE value for a day, D, was only positive if the considered hormone levels of two consecutive days, D and D-1, both showed a relative increase. Here, we applied the FIE to the day-specific E2 and E3G levels to compute day-specific FIE(E2) and FIE(E3G), respectively. This was the first time that day-specific E2 levels from individual cycles were available for FIE analysis.

Table 2 shows the results for FIE(E2) and FIE(E3G). There is a remarkable pattern, which serves to signal the entry into the fertile interval, the last day of the DF, and the day of DF collapse—a string of positive FIE(E2) values, a zero, and a negative—denoted as the identifying FIE(E2) sequence. The start of the identifying FIE(E2) sequence is Day −5 or Day −7 for cycles 1Y1, 2Y1, 4Y1, and 6Y1. No such identifying FIE(E3G) sequence was seen. There is a single positive FIE(E2) in the mid-follicular phase for 2Y1 on Day −8, but it could be easily dismissed as a false start to the fertile window since the next day was a 0 value. Ovulation, which occurred between the last morning of the DF and the first morning of DF collapse (Day −1 to Day 0), is clearly manifested by the transition from a positive string of FIE(E2) values to a 0 value (on Day −1) and then a negative FIE(E2) (on Day 0). Thus, FIE(E2) for these cycles is a means to track the ovulatory cycle and signal the crucial phases.

### 3.3. Application of the Area under the Curve (AUC) Algorithm with the Day-Specific E2, P and Day-Specific E3G, PDG Levels to Signal the Time-of-Cycle

**Figure 3:**
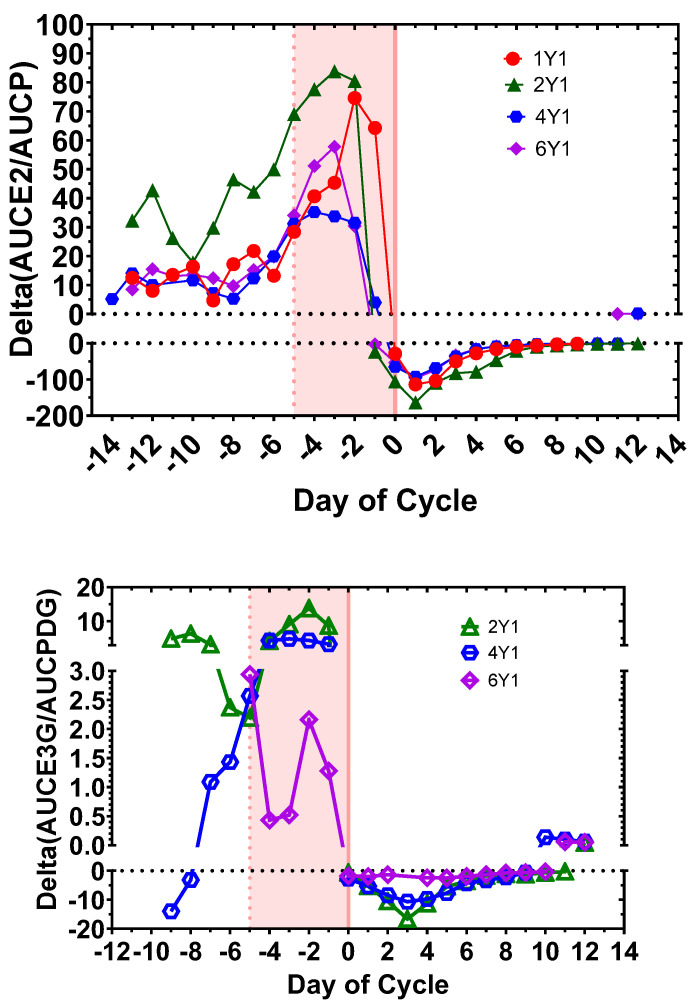
Comparison of Area Under the Curve (AUC) algorithm for mapping the time-of-cycle using the day-specific serum E2 and P versus urinary Mira^TM^ E3G and PDG for cycles 1Y1, 2Y1, 4Y1, and 6Y1 (**top**) and 2Y1, 4Y1, and 6Y1 (**bottom**). This algorithm is described in ref. [22] and is as follows: the AUC for E2 and P starting the first day of the menses or for E3G and PDG starting the first day after the end of menses were calculated. The day-specific ratios, AUCE2/AUCP and AUCE3G/AUCPDG, were calculated. The daily change in these ratios, the day-specific Delta (AUCE2/AUCP) and Delta (AUCE3G/AUCPDG), were then calculated as the metric for evaluating the time-of-cycle. In the case of the serum E2, P levels with the AUC algorithm, there is a striking drop to negative values on Day −1 (2Y1, 6Y1) and Day 0 (1Y1, 4Y1). There is a striking drop to negative values on Day 0 (2Y1, 4Y1, 6Y1) using the Mira^TM^ (E3G, PDG) levels as well. However, signaling of the predicted 6-day fertile interval (−5, 0; shaded) is only strong with the serum (E2, P) levels using the AUC algorithm. Day 0 is the day of dominant follicle (DF) collapse.

For the first time, day-specific (E2, P) data were available for the AUC algorithm [22]. The most striking result with the application of the AUC algorithm with both (E2, P) and (E3G, PDG) levels as variables was not only a sharp transition on Day −1 (2Y1 and 6Y1) and Day 0 (1Y1 and 4Y1) with (E2, P), but also a clear transition on Day 0 for (2Y1, 4Y1, and 6Y1) with (E3G, PDG) (Figure 3). This means that even with the fluctuations in urinary hormone levels registered by the Mira^TM^ monitor, the AUC algorithm clearly signals the day of transition to the luteal phase. The AUC algorithm provides greater resolution in terms of tracking this phase of the cycle with the Mira^TM^ monitor than did the PDG levels alone (see Figure 2).

However, the serum (E2, P) levels with the AUC algorithm are better at signaling the predicted 6-day fertile window compared to the (E3G, PDG) levels. There is a 1.57- to 2.14-fold increase in the Delta (AUCE2/AUCP) at Day −5, which could be useful in signaling the fertile window. There is much more variability in Delta (AUCE3G/AUCPDG) during the follicular phase (Figure 3).

### 3.4. Statistical Validation and Comparison of the FIE(E2) vs. FIE(E3G) Algorithms

For the following, in the *p*-value calculations that assess the probability of particular sequences occurring by chance alone (the null hypothesis), our reference models were random multinomial sequences with equal cell probabilities. The window of opportunity for the sequence to occur was factored into the calculation separately for each case. We assumed the cycles were independent, thus allowing for the multiplication of individual cycle probabilities in order to obtain an estimate for all cycles combined.

Under the null hypothesis that the identifying sequence (4–6 positives, 0, and a negative) was a random sequence, we modelled each outcome (or day) as a multinomial with three equally likely outcomes: positive, zero, and negative. The identifying sequence common to all four cycles consists of four positive values, then zero, and finally one negative value (Table 2). The probability that this sequence happens purely by chance is, therefore, (1/3)^6^. Now, since there are 13 possible days (i.e., the preovulatory–luteal transition interval) where this sequence or pattern can be inserted along a typical 28-day cycle, this probability was further divided by 13, resulting in a probability of approximately 1 in 9477 of each cycle exhibiting this pattern purely by chance. Thus, the ultimate probability of all four cycles acting this way during the preovulatory to luteal transition interval would raise that probability to the power of four, i.e., extremely unlikely to have occurred by chance alone. In the case of FIE (E3G), for these three cycles we simply took the complementary probability computed above, i.e., 1 minus (1/3)^6^ divided by 13 (since these cycles showed exactly the opposite behavior). For all three cycles to behave in this manner, we then arrived at an estimated probability of approximately 0.999. In other words, this behavior is very likely the result of chance alone, thus leading us to conclude that FIE with E3G does not exhibit the identifying pattern of FIE with E2.

### 3.5. Statistical Validation of the Delta (AUCE2/AUCP) and Delta (AUCE3G/AUCPDG) Algorithm

We modelled the characteristic shape of the Delta (AUCE2/AUCP) curve (Figure 3, top) as a sigmoid, positive to the left and then transitioning to negative to the right of the transition point. If we consider that the transition point is equally likely to occur at any 1 of the 23 cycle days, then the probability that it happens purely by chance either on Day −1 or 0 is 1/22. Thus, the probability all four cycles exhibit this transition pattern purely by chance is (1/22)^4^, or approximately 1 in 234,000. This is a highly significant *p*-value, leading to the conclusion that the observed scenario is extremely unlikely to have occurred by chance alone. The three Delta (AUCE3G/AUCPDG) cycles were modeled similarly (Figure 3, bottom). The characteristic curve is also a sigmoid, but only over 18 days (the common overlap for all cycles); positive for five consecutive values; and then negative for the remaining 13. Thus, the probability all three cycles exhibiting this pattern purely by chance is (1/18)^3^, or approximately 1 in 5800. We again conclude that the observed scenario is extremely unlikely to have occurred by chance alone.

## 4. Discussion

This is the first report of daily serum E2 and P levels from individual ovulatory cycles precisely indexed by the time of DF rupture. Furthermore, the daily E2 and P levels have been compared with the daily urinary E3G, PDG levels measured by the point-of-care (POC) fertility tracking Mira^TM^ monitor. The serum E2 levels showed greater accuracy and sensitivity in terms of signaling the start of the 6-day fertile window than E3G. Surprisingly, using a previously described Area Under the Curve (AUC) algorithm with (E2, P) or (E3G, PDG) as variables, both of these enabled the identification of the 24 h interval corresponding to [Day −1 (last day of DF) to Day 0 (first day of DF collapse)], the ovulatory/luteal transition point. This improves upon the existing fertility tracking technology and could have important implications especially in the realm of birth control by natural family planning/Fertility Awareness Based Methods (FABMs) [31,32,33].

These results beg the question: Can fingerstick blood be used for an (E2, P)-based fertility monitor? Technology for fingerstick blood as the sample to determine the POC levels of protein and other small molecules is already here. The concordance of fingerstick and venipuncture blood hormone levels has been rigorously shown [34]. There has been increasing sophistication in whole-blood-based lateral flow, microfluidic, and vertical flow assay systems for POC testing and diagnostics of various humoral and cellular biomarkers [27,34,35,36,37,38,39,40,41]. Presently, there is availability for fingerstick/whole blood POC measurements of fertility hormones: the SELFCHECK^TM^ pregnancy (hCG) fingerstick blood test can be purchased on Amazon.com, and the Getein 1100 Immunofluorescence Quantitative Analyzer is available as a POC platform for whole blood measurements of E2 and P (Getein Biotech, Inc., Nanjing, China). There is every expectation that there will be more choices for POC fingerstick E2 and P diagnostics in the future.

An important finding in this study was the usefulness of the Fertility Indicator Equation (FIE) with E2 as a variable and the AUC algorithm with (E2, P) and (E3G, PDG) as variables for fertility tracking. Here is a possible fertility tracking method with daily fingerstick (E2, P) levels for birth control: (a) fingerstick levels should be recorded starting calendar day (CD) 1; (b) P levels can be a constant check to distinguish preovulatory from luteal phases; (c) both FIE (E2) and Delta (AUCE2/AUCP) should be computed daily; (d) a positive FIE (E2) is the possible fertile start day and abstinence begins, but if the next day is 0 or negative, then no abstinence; (e) if a positive FIE (E2) string continues, it is the fertile window; (f) the FIE (E2) value of 0 that follows this string is Day −1; (g) the negative FIE (E2) value that follows is Day 0 (day of collapsed follicle); and (h) abstinence can end the day after. This can be corroborated by the concomitant AUC algorithm and a P rise. This should be readily interfaced with a suitable (E2, P) lateral flow or vertical flow assay device. Importantly, the AUC algorithm can be employed now with the Mira^TM^ fertility tracking system to more accurately signal the transition day from ovulation to the luteal phase.

The success of the proposed methodology for natural family planning/FABM birth control hinges on the sensitivity and precision of E2 and P measurements with a lateral or vertical flow POC assay. The serum E2 and P levels were determined with a high performance clinical platform, and there may be technical difficulties incorporating this power in a rapid POC strip. It should be noted that the FIE and AUC algorithms tested in this study are not exhaustive, others probably exist. And, although the four cycles involved over 100 blood draws and samples, the number of individual cycles is still limited. Subjects with a broader range of age and BMI and with cycles of more variable length in on-going studies will provide further evaluation of the conclusions reached from the present study.

## 5. Conclusions

There were greater fluctuations in the signature for the day-specific Mira^TM^ E3G and PDG levels compared to that for the day-specific serum E2 and P levels. Serum E2 was better at signaling the start of the 6-day fertile window using a mathematical algorithm, the Fertility Indicator Equation. However, another algorithm, the Area Under the Curve with (E2, P) or (E3G, PDG), showed that both serum and Mira^TM^ hormone measurements could pinpoint the 24 h interval of ovulation and transition to the luteal phase. The AUC (E3G, PDG) algorithm should be applicable to existing urinary hormone monitors. Serum E2 and P have promise as improved biomarkers for timing the major events during the menstrual cycle.

## 6. Patents

H.S.G., D.D.V. and S.J.U. have submitted a patent related to this manuscript.

## Figures and Tables

**Table 2:** Fertility Indicator Equation (FIE) values using day-specific serum E2 and urinary Mira^TM^ E3G levels for cycles 1Y1, 2Y1, 4Y1, and 6Y1 ^a^.

Day of Cycle Day 0 = 1st Day of DF Collapse	1Y1 FIE-E2	2Y1 FIE-E2	4Y1 FIE-E2	6Y1 FIE-E2	2Y1 FIE-E3G	4Y1 FIE-E3G	6Y1 FIE-E3G
−12	0	0	0	8.91			
−11	0	−0.25	−0.13	0			
−10	0	−3.65	0	0			
−9	−4.45	0	0	0			
−8	0	20.34	0	0	0	0	
−7	0	0	**5.11**	**14.22**	−3.39	−1.67	
−6	0	0	**2.66**	**3.99**	0	0	
−5	**27.66**	**13.85**	**6.36**	**15.97**	0	0	3.74
−4	**1.66**	**3.51**	**2.3**	**17.51**	0	0	0.89
−3	**0.35**	**2.2**	**7.05**	**6.87**	120.06	0	0
−2	**33.3**	**4.15**	**19.31**	**3.45**	26	0	0
−1	**0**	**0**	**0**	**0**	0	5.52	0
0	−**5.01**	−**22.73**	−**25.7**	−**39.41**	0	3.95	0
1	−19.4	0	−7.41	0	0	0	0
2	0	0	0	73.39	0	0	0
3	0	0	3.19	6.17	19.37	0	0
4	0	0	0.18	0	0	−0.188	0
5	0	0	0.35	0	−15.04	0	0
6	0	0	0	0	−5.62	0	0
7	0	0	−0.21	2.46	−7.75	0	−0.744
8	−14.37	0	0	2.86	0	0	0
9	−24.4	−2.22	0	0	0	−8.03	0
10		−3.95	−1.52	−1.28	0	0	−2.92
11		0	−6.58	−6.48	0	1.82	0
12		0	−9.71	−17.44	0	0	0

^a^ The Fertility Indicator Equation (FIE) is described in references [28,29]. The indeterminate day-specific FIE values are here denoted as ‘0’ rather than ‘indeterminate’. The bold FIE(E2) values in the green shading for cycles 1Y1, 2Y1, 4Y1, and 6Y1 indicate the identifying sequence of four to six consecutive positive values (start Day −5, Day −7), a transition to 0 on Day −1, and an ending negative value on Day 0. The start of the positive sequence signals the start of the predicted fertile interval (Day −5 to Day 0), and the identifying sequence reveals a highly fertile day (Day −1) and the day of DF collapse (Day 0). Note that FIE(E3G) did not work as a reliable indicator of the time-of-cycle. Day 0 values are indicated by gray shade.

## Data Availability

The data are available on request to the principal authors: S.J.U., D.D.V., and H.S.G.
